# Triglyceride glucose index for the detection of asymptomatic coronary artery stenosis in patients with type 2 diabetes

**DOI:** 10.1186/s12933-020-01108-2

**Published:** 2020-09-12

**Authors:** Pham Viet Thai, Hoang Anh Tien, Huynh Van Minh, Paul Valensi

**Affiliations:** 1 Department of Internal Medicine, Ninh Thuan Province General Hospital, Phan Rang - Thap Cham, Vietnam; 2 Department of Internal Medicine, Hue University of Medicine and Pharmacy, Hue University, Hue, Vietnam; 3 Unit of Endocrinology-Diabetology-Nutrition, Jean Verdier Hospital, APHP, Paris Nord University, CINFO, CRNH-IdF, Avenue du 14 Juillet, 93143 Bondy cedex, France

**Keywords:** Triglyceride glucose index, Coronary artery disease, Type 2 diabetes, Insulin resistance, Metabolic syndrome, Coronary computed tomography angiography

## Abstract

**Background:**

Triglyceride Glucose (TyG) index has been associated with an increased risk in cardiovascular events. Silent coronary disease is common in patients with type 2 diabetes. In Vietnam, a low-middle income country, the burden of cardiovascular disease is growing simultaneously with the epidemiologic transition. Our aim was to assess the prevalence of coronary stenoses (CS) in patients with type 2 diabetes and no history or symptom of cardiovascular disease and to investigate the association between TyG index and cardiovascular risk factors and both the presence and severity of CS. Futhermore, we assessed the value of TyG index in predicting subclinical CS.

**Methods:**

This was a cross-sectional observational study. We recruited 166 patients at Ninh Thuan General Hospital, Vietnam. TyG index and HOMA-IR were calculated, and a coronary computed tomography angiography (CCTA) was performed.

**Results:**

The population was classified according to tertiles of TyG index. The highest TyG values were associated with higher BMI, waist circumference, total cholesterol, LDL-cholesterol, triglycerides, plasma glucose, HbA1c levels and HOMA-IR, lower HDL-cholesterol, a higher incidence of metabolic syndrome and less frequent physical activity (p < 0.05 to < 0.0001). TyG index correlated with logHOMA-IR (p < 0.0001). CS ≥ 50% were present in 60 participants and 32 had coronary artery stenosis ≥ 70%. TyG index and HOMA-IR were significantly higher in patients with CS ≥ 70%. The number of narrowed coronary arteries and the degree of stenosis were associated with higher TyG index levels (p = 0.04 and < 0.005 respectively). A TyG index ≥ 10 was significantly associated with an increased risk of multiple coronary artery disease and of more severe CS. After adjusting for confounding factors, including logHOMA-IR, these risks remained mostly significant. A TyG index threshold at 10 resulted in 57% sensitivity and 75% specificity for predicting the presence of CS ≥ 70%. In subgroup analysis TyG index ≥ 10 was associated with an increased risk in CS ≥ 70% in patients treated with statin or antiplatelet therapy.

**Conclusion:**

More than one third of asymptomatic patients with type 2 diabetes had significant CS on CCTA. TyG index may be considered as a marker for insulin resistance and increased TyG index could identify patients with high risk of coronary artery stenoses and is associated with the number and the severity of artery stenoses.

## Background

Diabetes is one of the major risk factors for coronary artery disease [1]. It can be estimated that 20–30% of patients with coronary disease have known diabetes, and that an oral glucose tolerance test would diagnose diabetes or prediabetes in up to 70% of the other patients with coronary artery disease. Actually, patients with diabetes are at substantially increased risk of fatal and non fatal coronary events [2].

In people with diabetes, coronary disease is often silent, *i.e.* without any cardiac symptom. In asymptomatic patients with diabetes, the prevalence of silent myocardial ischemia as detected by a stress test varies, depending on several factors including the diagnostic test and whether or not cardiac imaging is used. Furthermore, the number of additional cardiovascular risk factors and the presence of diabetic complications, especially nephropathy and cardiac autonomic neuropathy play a role [3, 4]. The prevalence of silent ischemia which was 20–30% in the 1990’s [4] has dropped in the last decade [5]. Some recent studies showed that coronary computed tomography angiography (CCTA) may detect coronary artery stenoses in a large proportion of asymptomatic patients with diabetes [6, 7].

Insulin resistance is one of the pivotal risk factors for cardiovascular disease together with hyperglycemia, lipid disorders, hypertension and the production of clotting and inflammatory factors that may promote atherothrombotic changes. An independent association was reported between insulin resistance (assessed by HOMA-IR) and cardiovascular events both in subjects with and without diabetes [8–10]. In addition, several studies provide compelling evidence of an association between insulin resistance and subclinical vascular injury involving functional and structural damage to the arterial wall that is not explained by traditional risk factors [11]. Early atherosclerosis detected by intima-media thickness is also independently associated with insulin resistance in patients with type 2 diabetes [12]. Some studies have shown that the Triglyceride Glucose index (TyG) easily calculated by using fasting glucose and triglyceride levels may be used as a surrogate for insulin resistance [13–15]. In addition TyG index was shown to predict cardiovascular events [16] and to be a marker of subclinical atherosclerosis [17]. In a large population of individuals without conventional risk factors, TyG index appeared as an independent marker for predicting subclinical coronary artery disease defined as the presence of any plaque on coronary computed tomographic angiography (CCTA) [18]. A recent Korean study reported an association between TyG index value and an increased risk of coronary artery stenoses on CCTA in patients with type 2 diabetes [7]. However, this was not confirmed in another Korean study [19]. Therefore, there was a need for further studies assessing the role of insulin resistance in the occurrence of coronary artery stenoses and the severity of coronary artery disease and the usefulness of TyG index as a marker of subclinical coronary artery disease in patients with type 2 diabetes.

Vietnam is a low-middle income country, which is undergoing an important epidemiological transition. The prevalence of Vietnamese people with multiple comorbidities has been increasing [20] with a rapid rise in the overall morbidity and mortality from non-communicable diseases over the last two decades. The prevalence of silent coronary artery disease in the population with diabetes is expected to be greater than in higher income countries as a result of poorer control of cardiovascular risk factors.

The aim of this study performed in Vietnam was to assess the prevalence of coronary artery stenoses (CS) on CCTA and to investigate the association between TyG index and cardiovascular risk factors as well as the presence and severity of CS in patients with type 2 diabetes and no symptom or history of cardiovascular disease; the value of TyG index in predicting subclinical CS was also assessed.

## Methods

### Study population and design

This was a cross-sectional observational study. Enrolled participants were consecutive individuals diagnosed with type 2 diabetes according to the American Diabetes Association criteria [21], who were examined at Ninh Thuan General Hospital from April 2017 to May 2018. We excluded patients with type 1 diabetes and patients with an history or symptoms of cardiovascular disease such as myocardial infarction, congestive heart failure, arrhythmia or coronary revascularization, angina, edema or palpitations.

The study protocol was approved by the local ethic committee of Hue University of Medicine and Pharmacy. All patients provided written informed consent.

For each patient, medical history, physical activity, diabetes medications, treatment with statin or antiplatelet drugs was extracted from medical charts. Blood pressure was measured three times at approximately 2-min intervals after 5 min of seated rest, using an electronic sphygmomanometer (Omron HEM 1712), and mean values were calculated. Height without shoes was measured to the nearest cm using a portable stadiometer, and body weight was measured to the nearest 0.1 kg using a standard scale with patients wearing light indoor clothing and no shoes (Prestige, Hardik Medi Tech, India). Waist circumference was measured in the standing position, to the nearest cm, using a measuring tape (Prestige) placed mid-distance between the costal edge and the iliac crest.

### Biological measurements

Blood samples were collected after an overnight fast and analyzed in the hospital central laboratory. Biochemical assays were immediately processed. Plasma glucose, HbA1c, total cholesterol, triglycerides, HDL-cholesterol, and insulin were measured using HumaStar 600, Germany. LDL-cholesterol was calculated using Friedwald formula, and non-HDL cholesterol by subtracting HDL-cholesterol from total cholesterol. Glomerular filtration rate was estimated (eGFR) using CKD-EPI formula.

Two indicators of insulin resistance were calculated: TyG index =  Ln [Triglyceride (mg/dL) × Glucose (mg/dL)/2] [13] and homeostatic model assessment of insulin resistance (HOMA-IR) index (Insulin (mU/L) × plasma glucose (mmol/L)/22.5) [22]. Metabolic syndrome was diagnosed according to NCEP-ATP III criteria [23].

### Coronary computed tomography angiography imaging

Imaging was obtained by using a 64-slice scanner (Optima CT660, GE Healthcare, USA). According to the Society of Cardiovascular Computed Tomography 2014 guidelines [24], a 16-segment coronary artery tree model was used. All patients had normal sinus rhythm. If heart rate was faster than 70 bpm, an oral dose of 50–100 mg of metoprolol was administered 60–90 min before the study. If this did not lower the heart rate to the desired level, 5-mg doses of intravenous metoprolol were administered at 3- to 5-min intervals, up to a total dose of 15–30 mg. Images were obtained before and after administration of iodinated contrast (iopromide). Percent diameter stenosis was measured at the minimal lumen area (detected lumen contours), and corresponding reference diameter values were obtained from an automatic trend analysis of the vessel areas within the artery. The principles of coronary artery lesion interpretation included (1) systematic review of each coronary segment from multiple slices and in transverse sections, (2) awareness of relevant artifacts, and (3) assessment of stenosis severity using high resolution images (including multiplanar reformation format) in both longitudinal and transverse views of the artery. An image review in the frontal and lateral views could aid in the identification of artifacts. One specialist experienced in image reading reviewed the arterial tree in detail beginning in the axial (caudal) view since less processed trans-axial data are more robust. CS was considered as significant when ≥ 50% in one or more main branch of coronary arteries, moderate from 50 to 69% and severe if ≥ 70%.

### Statistical analyses

The sample size was calculated considering an estimated proportion of patients with significant coronary stenoses of 12% and a confidence level of 95%, leading to a minimum sample size of 162 patients.

The normality of the data was assessed using the Kolmogorov–Smirnov test. Results were expressed as mean ± SD, median (interquartile range) or percentages. Between group comparisons for quantitative parameters were performed using one-way ANOVA test or Kruskall-Wallis test. Between group comparisons for categorical parameters were performed by chi square tests. Correlations between quantitative parameters were performed by using Pearson test. ROC curve analysis was used to evaluate the accuracy of TyG index in detecting metabolic syndrome and CS. Multinomial logistic regression analyses were performed in order to assess the association of TyG index with CS after adjustment for confounding factors. Odds ratios with 95% confidence intervals (95% CI) for the risk of CS are reported. Subgroup analysis was performed after separating the patients according to statin and antiplatelet use. A p value < 0.05 was considered significant for all analyses. Statistical analyses were carried out using SPSS (Statistical Product and Services Solutions) software version 20.0.

## Results

### Main clinical characteristics of the study population

A total of 166 consecutive patients with type 2 diabetes were included: 62% male, mean age 58.9 ± 10.8 years, BMI 24.8 ± 2.6 kg/m^2^, HbA1c 7.9 ± 1.0%. TyG index mean value was 9.64 ± 0.63 (range: 7.80–10.96).

### Clinical and biochemical characteristics according to TyG index tertiles

We classified the population into tertiles of TyG index. In patients with higher TyG indexes, BMI, waist circumference, total cholesterol, triglycerides, LDL-cholesterol, non-HDL cholesterol, plasma glucose, HbA1c, insulin and HOMA-IR levels were higher whereas HDL-cholesterol levels and the frequency of physical activity were lower. There was no significant difference for the current treatments across TyG tertiles (Table 1).

**Table 1:** Clinical and biological characteristics according to the TyG tertiles

	Lowest tertile (7.80–9.37) (n = 56)	Mid tertile (9.38–9.99) (n = 55)	Highest tertile (10.00–10.96) (n = 55)	p value
Age (years)	58.5 ± 10.1	60.6 ± 11.4	57.5 ± 11.0	0.306
Gender (M/F)	36/20	34/21	33/22	0.897
BMI (kg/m^2^)	23.6 ± 2.8	25.0 ± 2.5	25.8 ± 2.1	< 0.001
Waist circumference (cm)	86.0 ± 11.7	91.2 ± 10.9	94.0 ± 9.9	0.001
Male	90.2 ± 11.9	94.9 ± 9.9	96.4 ± 9.1	0.041
Female	78.4 ± 6.1	85.2 ± 10.2	90.4 ± 10.2	< 0.001
Systolic blood pressure (mmHg)	138.9 ± 21.3	139.2 ± 22.9	143.7 ± 23.1	0.456
Diastolic blood pressure (mmHg)	85.9 ± 13.4	85.4 ± 14.1	85.6 ± 14.0	0.975
Duration of diabetes (years)	4 [3–6]	4 [3–8]	7 [3–12]	0.044
Practicing physical activity	24 (42.9%)	12 (21.8%)	7 (12.7%)	0.001
Smoking	15 (26.8%)	22 (40%)	24 (43.6%)	0.152
Metabolic syndrome	23 (41.1%)	42 (76.4%)	44 (80.0%)	< 0.001
Total cholesterol (mg/dL)	207.1 ± 46.3	224.1 ± 55.2	232.8 ± 52.7	0.03
Triglycerides (mg/dL)	146.9 ± 42.2	209.9 ± 44.2	330.8 ± 70.4	< 0.001
HDL-cholesterol (mg/dL)	50.0 ± 12.4	41.9 ± 8.2	40.1 ± 7.8	< 0.001
LDL-cholesterol (mg/dL)	126.1 ± 36.7	130.4 ± 44.0	146.0 ± 51.5	0.042
Non-HDL cholesterol (mg/dl)	157.1 ± 43.3	182.2 ± 54.6	192.7 ± 51.5	< 0.001
Fasting plasma glucose (mg/dL)	111.9 ± 27.7	159.7 ± 29.3	189.0 ± 43.2	< 0.001
HbA1c (%)	7.3 ± 0.6	8.0 ± 0.9	8.3 ± 1.0	< 0.001
TyG index	8.95 ± 0.37	9.67 ± 0.19	10.33 ± 0.23	< 0.001
Insulin (mU/L)	17 [12–28]	30 [18–39]	30 [18–39]	< 0.001
HOMA-IR index	4.45 [2.85–6.88]	10.20 [6.40–14.30]	12.40 [8.30–16.60]	< 0.0001
eGFR (mL/min/1.73 m^2^)	89.4 ± 28.1	82.2 ± 21.0	84.0 ± 20.3	0.239
Coronary artery stenosis ≥ 50% (%)	16 (28.6)	16 (29.1)	28 (50.9)	0.021
Coronary artery stenosis ≥ 70% (%)	5 (8.9)	11 (20.0)	16 (29.1)	0.026
Drug therapy				
Metformin	55 (98.2%)	53 (96.4%)	55 (100%)	0.359
Sulphonylurea	50 (89.3%)	51 (92.7%)	48 (87.3%)	0.634
Thiazolidinedione	1 (1.8%)	0 (0%)	0 (0%)	0.372
DPP-4 inhibitors	1 (1.8%)	1 (1.8%)	3 (5.5%)	0.432
α-glucosidase inhibitor	4 (7.1%)	12 (21.8%)	14 (25.5%)	0.029
Insulin	2 (3.6%)	0 (0%)	1 (1.8%)	0.369
Antiplatelet	30 (53.6%)	32 (58.2%)	32 (58.2%)	0.852
Statin	33 (58.9%)	32 (58.2%)	31 (56.4%)	0.961

Data are expressed as mean ± SD, median (interquartile range) or number (%)

Comparisons were performed using ANOVA or Kruskall-Wallis tests and chi square tests

*BMI* body mass index, *M/F* male/female, *eGFR* estimated glomerular filtration rate, *HOMA-IR* homeostatic model assessment of insulin resistance, *TyG* triglyceride glucose index

### Association of TyG index with metabolic syndrome and HOMA-IR index

**Fig. 1:**
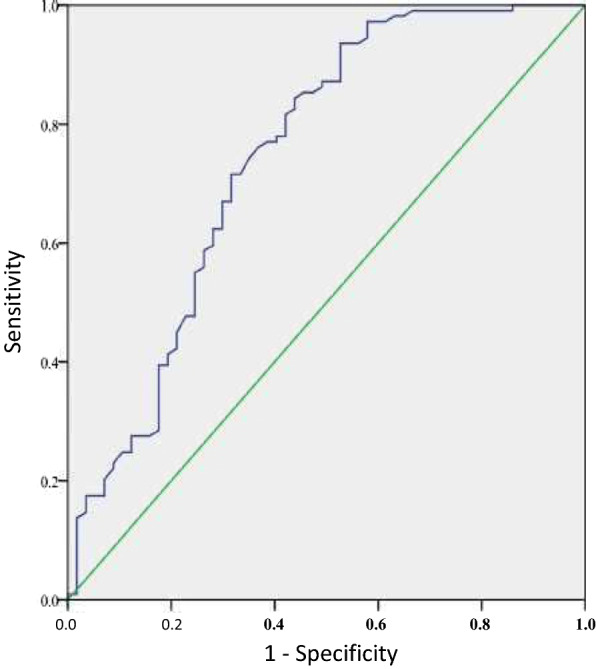
ROC curve of the use of TyG index in the detection of metabolic syndrome

**Fig. 2:**
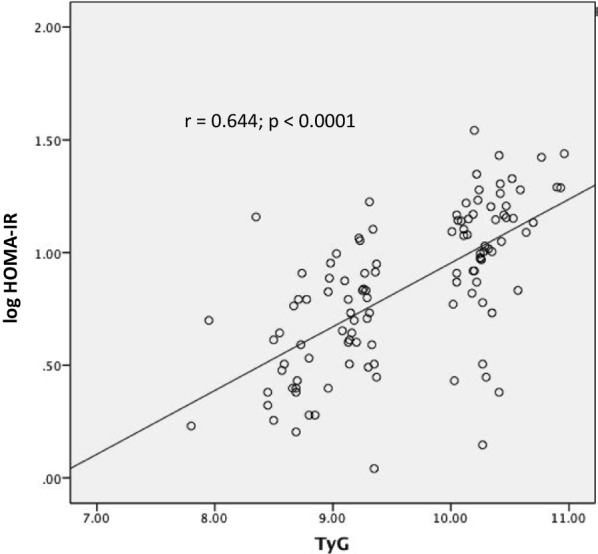
Correlation between TyG index and log HOMA-IR index

Metabolic syndrome was present in 109 patients (65.7%). The percentage of patients with metabolic syndrome was higher in the middle and high TyG index tertiles (Table 1). TyG index was significantly associated with metabolic syndrome with an area under the ROC curve at 0.745 (95% CI: 0.660–0.830, p < 0.001) (Fig. 1). A TyG index threshold at 9.145 resulted in a 93.6% sensitivity and a 52.6% specificity; positive and negative predictive values were respectively 77.3% and 79.4%. There was a significant correlation between TyG index and logHOMA-IR index (r = 0.644, p < 0.0001) (Fig. 2).

### Results of coronary computed tomography angiography and association with TyG index

In the total population (n = 166), 60 participants had CS ≥ 50%, including 32 with CS ≥ 70%. Twenty-one patients had 2- or 3-vessel disease (with stenosis ≥ 50%).

Compared to patients without significant CS, those with CS ≥ 50% were older, had longer diabetes duration, higher BMI, blood pressure and HbA1c, had more often been diagnosed with metabolic syndrome, were more frequently smokers, and were more often treated with statin or antiplatelet therapy. TyG index and HOMA-IR were significantly higher in patients with CS ≥ 70% (Table 2).

**Table 2:** Comparison between the patients without significant coronary artery stenosis (CS) or with moderate (50–69%) or severe (≥ 70%) CS

	Total	No significant CS	CS 50–69%	CS ≥ 70%	P
Number	166	106	28	32	
Age (years)	58.9 ± 10.8	57.2 ± 10.7	60.9 ± 9.8	62.8 ± 11.0	0.020
Gender (M/F)	103/63	63/43	21/7	19/13	0.301
BMI (kg/m^2^)	24.8 ± 2.6	24.5 ± 2.7	24.6 ± 2.5	26.0 ± 2.1	0.019
Waist circumference (cm)	90.4 ± 11.3	88.8 ± 11.3	92.1 ± 11.7	94.0 ± 10.4	0.054
Systolic blood pressure (mmHg)	140.6 ± 22.4	130.3 ± 18.5	152.0 ± 18.3	165.0 ± 11.1	< 0.0001
Diastolic blood pressure (mmHg)	85.7 ± 13.8	80.3 ± 11.2	91.1 ± 14.9	98.8 ± 9.5	< 0.0001
Duration of diabetes (years)	4.00 [3.00–8.00]	3.00 [2.00–4.00]	7.00 [6.00–9.00]	11.50 [8.00–12.00]	< 0.0001
Practicing physical activity	43 (25.9)	30 (28.3)	10 (35.7)	3 (9.4)	0.043
Smoking	61 (36.7)	27 (25.5)	15 (53.6)	19 (59.4)	< 0.0001
Metabolic syndrome	109 (65.7)	60 (56.6)	17 (60.7)	32 (100.0)	< 0.0001
Total cholesterol (mg/dl)	221.2 ± 52.3	222.7 ± 53.3	225.5 ± 46.1	212.5 ± 54.8	0.562
Triglycerides (mg/dl)	228.7 ± 93.4	218.3 ± 94.3	233.6 ± 98.6	259.0 ± 80.4	0.091
HDL-cholesterol (mg/dl)	44.0 ± 10.6	45.2 ± 11.3	44.2 ± 10.8	40.0 ± 6.4	0.053
LDL-cholesterol (mg/dl)	134.3 ± 45.0	130.5 ± 44.9	143.2 ± 51.5	139.2 ± 39.0	0.330
Non-HDL cholesterol (mg/dl)	177.2 ± 51.9	177.5 ± 52.7	181.3 ± 47.7	172.5 ± 54.0	0.804
Fasting plasma glucose (mg/dl)	153.6 ± 46.8	147.0 ± 43.5	148.2 ± 53.3	180.2 ± 43.0	0.001
HbA1c (%)	7.9 ± 1.0	7.6 ± 0.8	7.9 ± 0.8	8.9 ± 1.1	0.000
TyG index	9.64 ± 0.63	9.56 ± 0.61	9.58 ± 0.71	9.97 ± 0.49	0.005
Insulin (mU/L)	22 [15–34]	21 [14–35]	20 [12–31]	30 [20–40]	0.092
HOMA-IR index	8.10 [4.65–13.93]	7.60 [4.33–12.83]	6.20 [2.95–11.90]	13.45 [7.48–17.53]	0.001
eGFR (mL/min/1.73m^2^)	85.3 ± 23.5	88.7 ± 26.0	80.2 ± 15.8	78.3 ± 18.2	0.040
Drug therapy					
Metformin	163 (98.2)	104 (98.1)	28 (100)	31 (96.9)	0.660
Sulphonylurea	149 (89.8)	91 (85.8)	28 (100)	30 (93.8)	0.064
Thiazolidinedione	1 (0.6)	1 (0.9)	0 (0)	0 (0)	0.752
DPP-4 inhibitor	5 (3.0)	0 (0)	2 (7.1)	3 (9.4)	0.009
α-glucosidase inhibitor	30 (18.1)	5 (4.7)	9 (32.1)	16 (50.0)	< 0.0001
Insulin	3 (1.8)	0 (0)	2 (7.1)	1 (3.1)	0.034
Antiplatelet	94 (56.6)	46 (43.4)	23 (82.1)	25 (78.1)	< 0.0001
Statin	96 (57.8)	47 (44.3)	23 (82.1)	26 (81.2)	< 0.0001

Data are expressed as mean ± SD, median (interquartile range) or number (%)

Comparisons were performed using ANOVA or Kruskall-Wallis tests and chi square tests

*BMI* body mass index, *CS* coronary stenosis, *eGFR* estimated glomerular filtration rate, *HOMA-IR* homeostatic model assessment of insulin resistance, *M/F* male/female, *TyG index* triglyceride glucose index

The prevalence of significant CS did not differ across the tertiles of HOMA-IR (30.9, 32.1 and 45.5%, from the lowest to the highest tertile; p = 0.211). The prevalence of CS ≥ 50% and ≥ 70% were higher in the highest TyG index tertile subgroup *versus* the lowest and middle tertile subgroups (respectively, 50.9 *vs* 28.6 and 29.1%, p = 0.021; and 29.1 *vs* 8.9 and 20%, p = 0.026) (Table 1). TyG index significantly predicted the presence of CS ≥ 70% with an area under the ROC curve at 0.678 (95% CI: 0.582–0.775, p = 0.002). A TyG index threshold at 10 gave 57% sensitivity, 75% specificity, 56% positive predictive value and 75% negative predictive value.

**Fig. 3:**
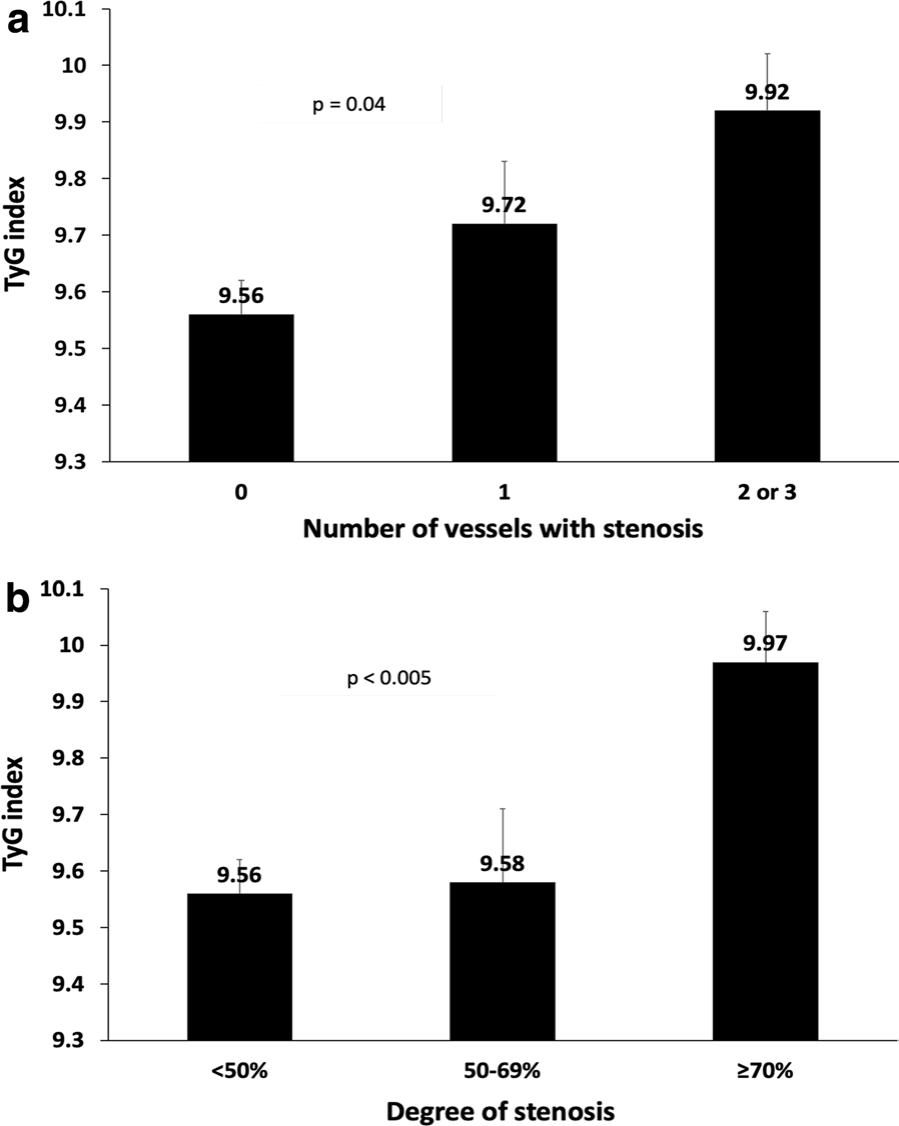
TyG index according to the number of coronary arteries with stenosis ≥ 50% (Part A) and the severity of coronary stenosis (Part B)

The number of stenosed coronary arteries (p = 0.04) and the degree of CS (p < 0.005) were positively associated with the TyG index level (Fig. 3a, b). TyG index ≥ 10 was significantly associated with an increased risk of having a higher number of stenosed arteries and more severe coronary artery stenoses. After adjusting for factors significantly associated with the severity of CS, including logHOMA-IR, these risks remained mostly significant (Table 3).

**Table 3:** Association between TyG index ≥ 10 and the number of coronary arteries with stenosis ≥ 50% and the degree of coronary stenosis

	Unadjusted OR (95% CI)	p value	Model 1 OR (95% CI)	p value	Model 2 OR (95% CI)	p value
Number of vessels with stenosis						
0	1		1		1	
1	3.77 (1.76–8.17)	0.001	4.95 (1.52–16.09)	0.008	6.88 (1.94–24.38)	0.003
2 or 3	3.90 (1.48–10.28)	0.006	3.02 (0.75–12.21)	0.121	2.74 (0.64–11.76)	0.176
Degree of coronary stenosis						
< 50%	1		1			
50–69%	2.93 (1.24–6.92)	0.014	4.86 (1.39–17.03)	0.013	6.89 (1.80–26.44)	0.005
≥ 70%	4.88 (2.11–11.28)	0.0001	4.36 (1.15–16.53)	0.030	4.04 (1.00–16.34)	0.050

Multinomial logistic regression analyses were performed

*Model 1* Adjusted for duration of diabetes of 4 years (median value in the total population), body mass index, estimated glomerular filtration rate, practicing physical activity, smoking, HbA1c of 7.9% and systolic blood pressure of 140 mmHg (mean values in the total population), *Model 2* Model 1 + adjusted for logHOMA-IR, *OR* odds ratio, *CI* confidence interval

**Fig. 4:**
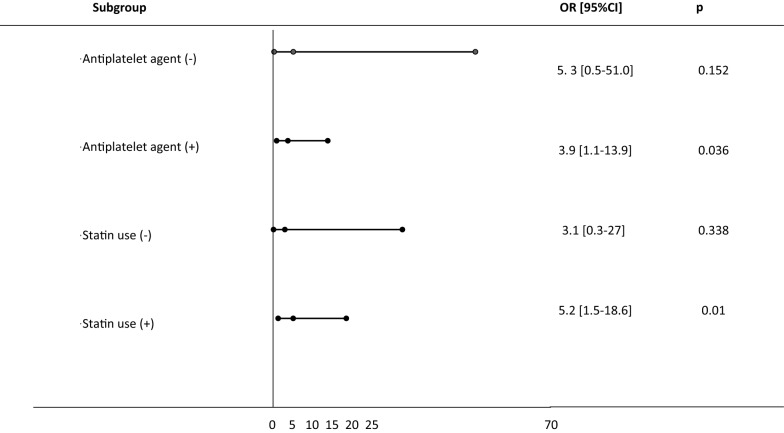
Subgroup analysis for the risk of coronary stenosis ≥ 70%. Odds ratio of TyG index ≥ 10 compared to < 10

Subgroup analyses showed that TyG index ≥ 10 was associated with an increased risk of CS ≥ 70% in patients on statin or antiplatelet therapy but not in those not taking these treatments (Fig. 4).

## Discussion

In this study we included patients with type 2 diabetes and no symptom or history of cardiovascular disease but with rather poor control of hypertension and lipid disorders. TyG index correlated with HOMA-IR. Patients with higher TyG index had more metabolic disorders. More than one third of our patients had coronary stenoses on CCTA and 19% had severe CS (≥ 70%). Patients with severe CS had higher TyG index and HOMA-IR. The prevalence of CS ≥ 50% and ≥ 70% was higher in the high tertile of TyG index but did not differ across the tertiles of HOMA-IR. TyG index significantly predicted the presence of CS ≥ 70%. In addition, we showed for the first time in an asymptomatic population that the number of stenosed coronary arteries and the degree of coronary stenosis were associated with high TyG index levels. TyG index ≥ 10 was significantly associated with a 3- to 5-fold greater risk of higher number of stenosed coronary arteries and of more severe coronary stenoses, and these risks remained mostly significant after adjustment for confounding factors. Our results suggest that in patients with type 2 diabetes TyG index may be considered as a marker of insulin resistance and could identify patients with high coronary risk.

### TyG index as a marker of insulin resistance and metabolic syndrome

TyG index, a composite indicator based on triglyceride level and fasting plasma glucose value, was shown to correlate with insulin resistance as assessed by hyperinsulinemic euglycemic clamp or HOMA-IR [13–15] and may thus be used as a surrogate marker of insulin resistance. When compared to clamp, TyG index could even perform better than HOMA-IR [25]. TyG index was also reported to be a marker of metabolic disorders [7, 14, 26] and a good predictor of incident type 2 diabetes [27–29]. In our population of patients with type 2 diabetes, higher TyG index was strongly associated with higher HOMA-IR and more pronounced metabolic disorders including higher BMI, increased waist circumference, total cholesterol, triglycerides, LDL-cholesterol, non-HDL cholesterol, plasma glucose and HbA1c levels, lower HDL-cholesterol levels and also less physical activity. This index was not associated with current glucose-lowering treatments except for α-glucosidase inhibitors (only 30 patients on this treatment) nor with lipid lowering treatment, noting that none of our patients was on fibrate, which might have altered TyG index. Interestingly, TyG index calculation effectively led to metabolic syndrome diagnosis with rather good performances when using a threshold at 9.145.

### TyG index as a marker of atherosclerosis and cardiovascular complications

Several studies suggest that TyG index might be recognized as a risk factor for cardiovascular complications and a marker of atherosclerosis. TyG index was shown to be an independent predictor of cardiovascular events in a healthy population [16] and to be associated with a higher incidence of cardiovascular outcomes in patients with stable coronary artery disease [30] including those with type 2 diabetes [31], in patients with non-ST elevation acute coronary syndrome [32] and in those with acute ST-elevation myocardial infarction after percutaneous coronary intervention [33]. This index was reported to be a better marker than HOMA-IR for subclinical carotid atherosclerosis and arterial stiffness in the general population [34–36] and in lean postmenopausal women [17]. In a healthy population TyG index was more independently associated with coronary artery calcifications than was HOMA-IR [26]. Elevated TyG index was also shown to be an independent predictor of progression of coronary calcifications [37].

### Cardiovascular risk in contemporary South-Asian countries

Cardiovascular disease is becoming a major cause of deaths in Vietnam like in other low-middle income countries. In the setting of acute myocardial infarction, the incidence of in-hospital death rates is higher in patients with multiple cardiac comorbidities [38] with a need to improve guideline adherence [39]. The proportion of patients with diabetes and silent coronary artery disease is expected to be greater than in higher income countries. In the present study, CCTA detected coronary stenoses ≥ 50 in 36% and ≥ 70 in 19% of the patients. In two previous studies which similarly performed CCTA in asymptomatic individuals, the proportion of patients with CS ≥ 70% was found lower: 6.3% in a US population of patients with type 1 or type 2 diabetes considered to be at high cardiovascular risk [6] and 12.3% in a Korean population of patients with type 2 diabetes [7]. Compared to the latter study, our population had some similarities but the risk was greater because blood pressure, triglycerides and LDL-cholesterol levels were higher, and TyG index was also higher. The levels of these risk factors were clearly above target values according to the recent guidelines [1].

### TyG index is an independent marker of the severity of silent coronary disease

In our study, TyG index was associated with an increased risk of CS on CCTA, consistently with previous reports [7]. Additionnally, since diabetes is associated with an increase in the extension and severity of coronary artery disease, we evaluated for the first time the severity of CS and found that TyG index ≥ 10 was associated with an increased number of stenosed vessels and more severe stenosis. This risk was independent of diabetes duration, BMI, systolic blood pressure, HbA1c, eGFR, physical activity and smoking. This result is consistent with a previous study in patients with non-ST elevation acute coronary syndrome observing an independent association of TyG index with the number of stenosed coronary arteries and the SYNTAX score [32]. This suggests that insulin resistance as expressed by TyG index may contribute to atherosclerosis in addition to cardiovascular risk factors.

However the association of TyG index with more severe coronary disease remained significant after further adjustment on logHOMA-IR, suggesting that both metabolic disorders expressed by TyG index may play a role in coronary atherosclerosis. Improved glycemic control is expected to reduce TyG index level and may contribute to reduce cardiovascular outcomes in patients with type 2 diabetes [40]. Whether or not triglycerides are involved in the atherosclerotic process is still debated. Some data strongly suggest a relation between elevated triglyceride levels and cardiovascular disease [41, 42] with a residual cardiovascular risk associated with triglycerides in patients with type 2 diabetes who have reached their LDL-cholesterol target [43, 44]. A major role of the retention of cholesterol-rich and triglyceride-rich apoB-containing remnants within the arterial wall is to be considered in the pathogenesis of atherosclerosis [45].

In subgroup analyses we observed that TyG index was associated with an increased risk of CS ≥ 70% in patients on statin or antiplatelet therapy. This suggests that TyG index might account at least partly for the residual risk that remains despite these treatments. Thus, triglyceride level lowering appears as an additional target in patients at high cardiovascular risk [1, 43, 46–49].

Our study has some limitations. First, its cross-sectional design prevents us from considering the results as a definite causality relationship and the mechanisms behind the association of TyG index and CS remain to be clarified. Second, our study population was relatively small. A TyG index threshold at 10 to identify patients at high risk of severe silent coronary stenosis needs to be confirmed in further studies. Third, we compared TyG index with HOMA-IR and not with the results of an hyperinsulinemic euglycemic clamp, the gold standard method for measuring insulin resistance. However, several studies have previously shown similar associations of TyG index with clamp test results. Fourth, we did not record any nutritional data and therefore, we were unable to adjust for diet habits that can dramatically change triglyceride levels. Fifth, the participants were recruited in one hospital center, which may limit the generalizability of our results. However, our study has a major strength, as we performed CCTA in a relatively homogeneous population of patients with type 2 diabetes who did not have any evidence of cardiovascular disease.

## Conclusion

Our results strongly suggest that in patients with type 2 diabetes, TyG index may be used as a tool to assess insulin resistance and to identify patients with metabolic syndrome. This simple and inexpensive tool may be easily used in low-middle income countries.

In our population, more than one third of the participants had CS on CCTA and TyG index was better than HOMA-IR to identify patients at high risk. Higher TyG index was associated with the number of stenosed coronary arteries and the severity of stenosis independently from classical risk factors. The predictive value of TyG index seems to be additional to classical risk factors and remains significant even in patients on statin or antiplatelet therapy. Insulin resistance but also high triglyceride levels might thus be involved in the residual cardiovascular risk. Finally, TyG index might be beneficial for stratification and intervention to prevent major cardiac events. Further studies in larger populations are needed to assess the importance of these results.

## Data Availability

The datasets generated during and/or analysed during the current study are available from the corresponding author on reasonable request.

## Abbreviations

CCTA: Coronary computed tomography angiography
CS: Coronary stenoses
eGFR: Estimated glomerular filtration rate
HOMA-IR: Homeostatic model assessment of insulin resistance
TyG: Triglyceride Glucose index
